# Lysosomotropic agents as HCV entry inhibitors

**DOI:** 10.1186/1743-422X-8-163

**Published:** 2011-04-12

**Authors:** Usman A Ashfaq, Tariq Javed, Sidra Rehman, Zafar Nawaz, Sheikh Riazuddin

**Affiliations:** 1 Division of Molecular Medicine, National Centre of Excellence in Molecular Biology, University of the Punjab, Lahore, Pakistan; 2 Braman Family Breast Cancer Institute, University of Miami, USA; 3 Allama Iqbal Medical College, University of Health sciences, Lahore, Pakistan

## Abstract

HCV has two envelop proteins named as E1 and E2 which play an important role in cell entry through two main pathways: direct fusion at the plasma membrane and receptor-mediated endocytosis. Fusion of the HCV envelope proteins is triggered by low pH within the endosome. Lysosomotropic agents (LA) such as Chloroquine and Ammonium chloride (NH_4_Cl) are the weak bases and penetrate in lysosome as protonated form and increase the intracellular pH. To investigate the antiviral effect of LA (Chloroquine and NH_4_Cl) on pH dependent endocytosis, HCV pseudoparticles (HCVpp) of 1a and 3a genotype were produced and used to infect liver cells. The toxicological effects of Chloroquine and NH_4_Cl were tested in liver cells through MTT cell proliferation assay. For antiviral screening of Chloroquine and NH_4_Cl, liver cells were infected with HCVpp of 3a and 1a genotype in the presence or absence of different concentrations of Chloroquine and NH4Cl and there luciferase activity was determined by using a luminometer. The results demonstrated that Chloroquine and NH_4_Cl showed more than 50% reduction of virus infectivity at 50 μM and 10 mM concentrations respectively. These results suggest that inhibition of HCV at fusion step by increasing the lysosomal pH will be better option to treat chronic HCV.

## Introduction

Hepatitis C Virus (HCV) is an enveloped, positive stranded RNA virus classified in the family of Flaviviridae. HCV causes acute and chronic hepatitis infection which can eventually lead to permanent liver damage, hepatocellular carcinoma and death. It is estimated that three to four million people are infected with HCV every year [1]. HCV genome encodes a single polyprotein precursor of just over 3000 amino acids. This polyprotein precursor is co- and posttranslationally processed by cellular (signal peptidase and signal peptide peptidase) and viral (NS2-3 and NS3-4A) proteases to yield four structural (Core, E1, E2 and P7) and six non structural (NS2, NS3, NS4A, NS4B, NS5A, NS5B) proteins. HCV envelope is formed by E1 and E2 glycoprotein heterodimers which are essential for virus entry into cells [2]. HCV E1 and E2 fusion was enhanced at low pH, suggesting that HCV enters cells via the endosomal pathway and that E1 and/or E2 undergo conformational modifications that allow fusion of viral and cellular membranes [3].

There are six major and more than 80 subtypes of HCV. This classification is based on nucleotide variation among different HCV isolates. They occur in different proportion in different parts of the world. Genotype 1a and 1b are the most common genotypes in the United States and Europe [4,5]. The most prevalent HCV genotype in Pakistan is 3a followed by 3b and 1a [6].

Presently, there is no vaccine available for prevention of HCV infection due to high degree of strain variation. Current therapeutic options for hepatitis C are limited, especially for genotype 1. For genotypes 2 and 3, pegylated interferon in combination with ribavirin, can lead to a sustained virological response in up to 80% of patients [7]. However, this therapy is expensive and often associated with side effects that may lead to discontinuation of therapy [8]. Hemolytic anemia, cough, shortness of breath & treatogenicity are the most common adverse effect associated with ribavirin treatment, and muscle aches, fatigue & neuropsychiatric adverse effects of IFN-α lead to premature cessation of therapy in 10 to 20% of patients [9,10]. Moreover, cost of interferon for 6 month treatment ranging from PKR 50,000 to 150,000 is beyond the financial range of most patients. Hence, there is a need to develop anti HCV agents, which are less toxic, more efficacious and cost-effective.

The term "lysosomotropic agent" was introduced by DeDuve and co-workers (1974) to designate substances that are taken up selectively into lysosomes. This definition leaves open the chemical nature of a lysosomotropic substance and the mechanism of its uptake. Lysosomotrpic agents such as NH_4_C1, chloroquine and methylamine penetrate acidic compartments of the cell and accumulate as protonated forms, resulting in an increase in the intravesicular pH [11-13].

Chloroquine, which is widely used for the treatment of malaria, is a well-established inhibitor of autophagic proteolysis which acts by inhibiting acidification of lysosomes and endosomes [14]. It has been reported that lysosomotropic agents such as Chloroquine and NH_4_Cl exert direct antiviral effects on several RNA viruses including coronaviruses, flaviviruses and human immunodeficiency virus (HIV) [15-18]. Moreover, clinical studies have demonstrated the safety, tolerability, and efficacy of lysosomotropic agents in the antiviral treatment of HIV infection [19,20].

In the current study HCV entry is blocked by Lysosomotropic agents. Firstly toxicological analysis of Chloroquine and NH_4_Cl were done in liver cells. After toxicological analysis, antiviral effects were studied in HCVpp of 1a and 3a genotype.

## Materials and methods

### Cell lines

Huh-7 and HEK 293 T cells were cultured in Dulbecco's Modified Eagle medium (DMEM) supplemented with 10% fetal calf serum, 100 IU/ml penicillin and 100 μg/ml streptomycin, at 37°C in an atmosphere of 5% CO_2_. Huh-7 was kindly provided by Dr. Zafar Nawaz (Biochemistry and Molecular Biology Department, University of Miami, USA.

### Plasmids

The pcDNA-E1E2 expression vector encoding the E1 and E2 glycoproteins (171-746) of HCV genotype 3a and 1a, was generated by inserting into a nonpackageable, CMV promoter-driven expression construct. The CMV-Gag-Pol murine leukemia virus (MLV) packaging construct, encoding the MLV *gag *and *pol *genes, and the pTG-Luciferase plasmid provided by Dr Jaean Dubison, France.

### Production of HCVpp and infection: Production of HCVpp and infection

HCVpp were produced by co-transfection of 293-T cells with equal amounts of three expression vector as described previously [21]. Supernatants containing HCVpp were harvested 48 h later, filtered through 0.45 μm pore-sized membranes and stored at -80°C before use in infection of Huh7 cells.

### Cell proliferation assay

MTT (3-[4, 5-dimethylthiazol-2-yl]-2, 5-diphenyltetrazolium bromide) is a rapid and sensitive *in-vitro *procedure for evaluating cellular toxicity of compounds. The MTT substance is reduced by mitochondrial succinic dehydrogenases in living cells to purple formazan crystals that are not soluble in aqueous water. The absorption of dissolved formazan in the visible region correlates with the number of viable cells [21]. To investigate cellular toxicity, 2 × 10^4 ^Huh-7 cells were plated into 96-well plates. After 24 h, different concentrations of Chloroquine and NH_4_Cl were added and the plate was sealed and kept at 37°C in an atmosphere of 5% CO_2 _for 24 h. After 24 h, fresh media (100 μl) and MTT solution (5 mg/ml in PBS) were added to all wells in Columns 1-11. Wrapped the plate in aluminium foil and incubated for 3-4 h at 37°C. Media was carefully removed and added 100 μl of DMSO to dissolve the formazan crystals in Columns 1-11. MTT formazan product was determined by measuring absorbance with an enzyme-linked immunosorbent assay (ELISA) plate reader at a test wavelength of 570 nm and a reference wavelength of 620 nm.

Cell viability was obtained using the following equation.

### Antiviral analysis of Chloroquine and NH_4_Cl

To investigate anti-fusion effect of Chloroquine and NH_4_Cl, Huh-7 cells were incubated in the presence or absence of Chloroquine and NH_4_Cl at 37°C for 30 min. After 30 min Huh-7 cells were infected with HCVpp of 3a and 1a genotype in the presence or absence of different concentrations of Chloroquine and NH_4_Cl and incubated for additional 24 h. After 24 h cells were lysed and luciferase activity was determined by using a luminometer.

### Statistical Analysis

All statistical analysis was done using SPSS software (version 16.0, SPSS Inc). Data are presented as mean ± SD. Numerical data were analyzed using student's t-test and ANOVA. P value < 0.05 was considered statistically significant.

## Results

### MTT Cell Proliferation Assay

**Figure 1:**
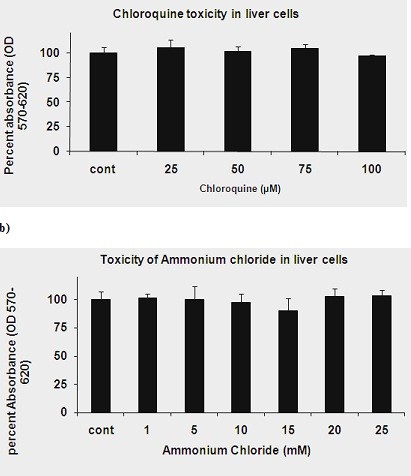
**Toxicological study of Chloroquine and NH_4_Cl in Huh-7 cells**: Huh-7 cells were plated at the density of 2 × 10^4 ^in 96 well plates. After 24 h cells were treated with different concentrations of chloroquine and NH_4_Cl and control consisted of solvent in which compound dissolved. After 24 h incubation period add MTT solution to all wells and incubated for 3-4 h at 37°C. Viable cells convert MTT to purple formazan crystal. Added DMSO to dissolve the formazan crystals and read absorbance at 570 nm and 620 nm. (a) Toxicological analysis of Chloroquine in Huh-7 cells through MTT cell proliferation assay. (b) Toxicological analysis of NH_4_Cl in Huh-7 cells through MTT cell proliferation assay.

MTT assay is a widely used test for evaluating cytotoxicity of compounds/herbs in cell cultures. The MTT substance is reduced by mitochondrial succinic dehydrogenases in living cells to purple formazan crystals that are not soluble in aqueous water. The absorption of dissolved formazan in the visible region correlates with the number of alive cells [21]. Cytotoxic effects of Chloroquine and NH_4_Cl were analyzed after 24 h incubation of Huh-7 cells with different concentrations of compounds. Figure 1 showed that cell proliferation of liver cells is unaffected at high concentration. After toxicological analysis through MTT proliferation assay, antiviral activities of Chloroquine and NH_4_Cl were tested at non toxic concentrations.

### HCVpp infection is pH dependent

**Figure 2:**
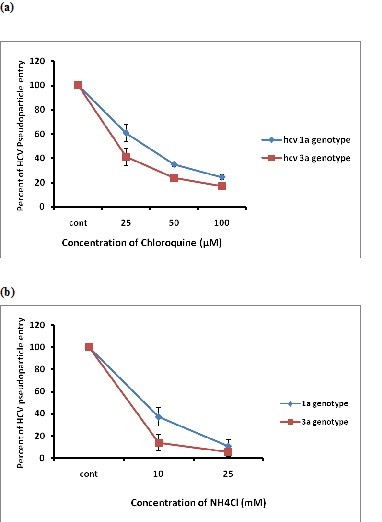
**Dose-dependent inhibition of HCVpp of 3a and 1a genotype with lysosomotropic agents**. HCVpp were produced in HEK 293 T cells and collected in media after filtration in 0.45 micron filter. Huh-7 cells were incubated in the presence or absence of Lysosomotropic agents such as Chloroquine and NH_4_Cl at 37°C for 30 min. After 30 min Huh-7 cells were infected with HCVpp of 3a and 1a genotype in the presence or absence of different concentrations of lysosomotropic agents and incubated for additional 24 h. After 24 h cells were lysed and luciferase activity was determined by using a luminometer. Luciferase activity is not reported as an absolute value, but is calculated relative to the 'no drug' condition and reported on the *y*-axis as a percentage. Results are represented as the average and standard error for three independent experiments. (a) Dose-dependent inhibition of Chloroquine against HCVpp of 1a and 3a genotype. (b) Dose-dependent inhibition of NH_4_Cl against HCVpp of 1a and 3a genotype. P value > 0.05 vs control was considered as statistically significant.

Enveloped viruses enter cells through two main pathways: direct fusion at the plasma membrane and receptor-mediated endocytosis. Fusion of the viral envelope protein(s) is triggered by low pH within the endosome. Lysosomotropic agents such as Chloroquine and NH_4_Cl, have been used to demonstrate the pH sensitivity of virus entry. We therefore tested the infectivity of HCVpp after treatment of target cells with different concentrations of Chloroquine and NH_4_Cl. HCVpp of 1a and 3a genotype demonstrated dose- dependent inhibition in the presence of Chloroquine and NH_4_Cl. Chloroquine and NH_4_Cl showed greater than 50% reduction of virus infectivity at 50 μM and 10 mM concentration respectively, suggesting a pH-sensitive route of virus entry (Figure 2a and 2b).

## Discussion

HCV entry is a multistep process requiring four cellular receptor, fusion and endocytosis. HCV fusion depends on E1 and E2, viral dose, and occurs within a specific pH range. Targeting pH dependent endocytosis is a useful tool to identify antiviral drugs against. A major advancement to look into HCV entry process was the development of HCVpp, consisting of native HCV envelope glycoproteins, E1 and E2, assembled onto retroviral core particles [3,22,23]. This system is potentially very powerful tool to identify and characterize molecules that block HCV entry. In this study, HCVpp of local HCV genotype 3a and 1a were produced to study early entry steps mediated by HCV envelope glycoproteins. This assay is based on the quantification of retroviral DNA synthesis, which occurs soon after the fusion of the retroviral particle with a cellular membrane. Presumably, this assay is only dependent on the entry steps mediated by the heterodimer E1E2 (binding, endocytosis, and fusion) and on the activity of the reverse transcriptase of the HCVpp retroviral core.

**Figure 3:**
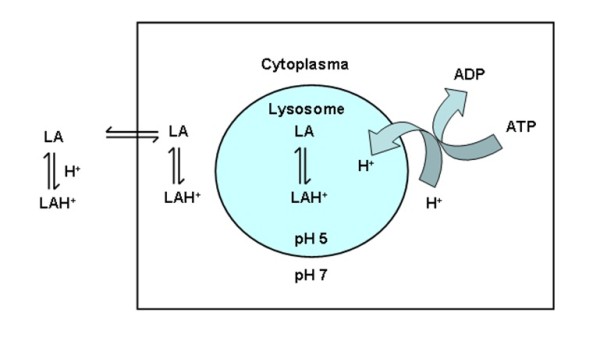
**Schematic model of lysosomal pH maintenance and intrasomal trapping of lysosomal agents (LA) or weak bases**.

The intracellular sub-compartments such as lysosomes and endosomes have an acid nature with pH of about 5 [14]. Lysosomotropic agents such as Chloroquine and NH_4_Cl are weak bases which have a tendency to accumulate in these compartments. Lysosomotropic agents are captured by protonation inside the lysosomes and accumulate there (Figure 3). The ratio of intra/extra lysosomal concentrations of these substances is equal to the ratio of concentration of hydrogen ions in lysosomes and in their vicinity, i.e. 1 : 100 if we suppose that pH in lysosomes is 5 and in cytoplasm 7. The amount of the permeable form of lysosomotropic agents passing through the membrane depends on the substance pKa value and pH value of solution. The higher the pKa value, the lower the permeable form ratio. This has led to the assumption that the specific uptake of lysosomotropic substances into lysosomes depends on the acidic pH of these compartments, ion-trapping weak bases [13]. In this model, the most suitable weak bases are those with a pKa around 8.

**Figure 4:**
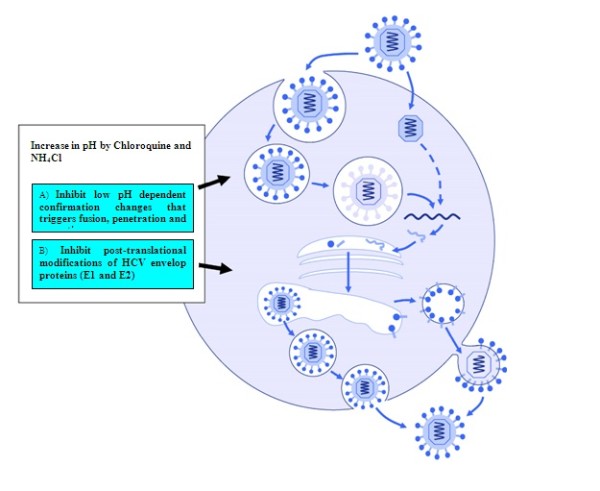
**Schematic representation of Inhibition of HCV by Increase the pH through Chloroquine and NH_4_Cl**.

NH_4_Cl and Chloroquine are lysosomal weak bases that are known to affect acid vesicles leading to dysfunction of several proteins. Previous studies of Chloroquine and NH_4_Cl have demonstrated that it has multiple effects on mammalian cells in addition to the elevation of endosomal pH, including the prevention of terminal glycosylation of immunoglobulin's [24]. When added to virus-infected cells, chloroquine inhibited later stages in vesicular stomatitis virus maturation by inhibiting the glycoprotein expression at the cell surface [25], and it inhibited the production of infectious HIV-1 particles by interfering with terminal glycosylation of the glycoprotein [26,27]. Increase in pH by Chloroquine and NH_4_Cl, firstly inhibits low pH confirmational changes that triggers fusion, penetration and uncoating and secondly inhibits posttranslational modification of HCV enveloped proteins (E1 and E2) (Figure 4). Our results demonstrated that, Chloroquine and NH_4_Cl inhibit HCVpp entry in a dose-dependent manner at non toxic concentration. Chloroquine and NH_4_Cl resulted in greater than 50% reduction of virus infectivity at a concentration of 50 μM and 10 mM respectively, suggesting a pH-sensitive route of virus entry (Figure 2a and 2b).

In conclusion, inhibition of HCV entry by increasing the pH through lysosomotropic agents is an important target to identified new drugs against HCV and combination of lysosomotropic agents with interferon will be better option to treat chronic HCV.

## Abbreviations

**HCV**: Hepatitis C virus; **LA**: Lysosomotropic agents; **Huh-7**: Human Hepatoma Cell line.

## Competing interests

The authors declare that they have no competing interests.

## Authors' contributions

UAA, TJ and SDR contributed equally in lab work and manuscript write up. ZN and SRD were the principal investigator and provide all facilities to complete this work. All the authors read and approved the final manuscript.

## Authors' information

Usman Ali Ashfaq (PhD Molecular Biology), Imran shahid (M Phil Molecular Biology), Tariq Javed (M. Phil pharmaceutical chemistry, Sidra Rehman (MSc Chemistry) and Sheikh Riazuddin (PhD molecular Biology and Dean Post graduate study at Allama Iqbal medical college, Lahore

## References

[B1] Thomson BJ Finch RG Hepatitis C virus infection Clin Microbiol Infect 2005 11 86 94 10.1111/j.1469-0691.2004.01061.x 15679481

[B2] Flint M McKeating JA The role of the hepatitis C virus glycoproteins in infection Rev Med Virol 2000 10 101 117 10.1002/(SICI)1099-1654(200003/04)10:2<101::AID-RMV268>3.0.CO;2-W 10713597

[B3] Hsu M Zhang J Flint M Logvinoff C Cheng-Mayer C Rice CM McKeating JA Hepatitis C virus glycoproteins mediate pH-dependent cell entry of pseudotyped retroviral particles Proc Natl Acad Sci USA 2003 100 7271 7276 10.1073/pnas.0832180100 12761383 PMC165865

[B4] McOmish F Yap PL Dow BC Geographical distribution of hepatitis C virus genotypes in blood donors: an international collaborative survey J Clin Microbiol 1994 32 884 892 7913097 10.1128/jcm.32.4.884-892.1994 PMC263157

[B5] Nousbaum JB Pol S Nalpas B Group atCS Hepatitis C virus type 1b (II) infection in France and Italy Ann Intern Med 1995 122 161 168 7810932 10.7326/0003-4819-122-3-199502010-00001

[B6] Idrees M Riazuddin S Frequency distribution of hepatitis C virus genotypes in different geographical regions of Pakistan and their possible routes of transmission BMC Infect Dis 2008 8 69 10.1186/1471-2334-8-69 18498666 PMC2409346

[B7] Flamm SL Chronic hepatitis C virus infection Jama 2003 289 2413 2417 10.1001/jama.289.18.2413 12746366

[B8] Cornberg M Wedemeyer H Manns MP Treatment of chronic hepatitis C with PEGylated interferon and ribavirin Curr Gastroenterol Rep 2002 4 23 30 10.1007/s11894-002-0034-y 11825538

[B9] Khakoo S Glue P Grellier L Wells B Bell A Dash C Murray-Lyon I Lypnyj D Flannery B Walters K Dusheiko GM Ribavirin and interferon alfa-2b in chronic hepatitis C: assessment of possible pharmacokinetic and pharmacodynamic interactions Br J Clin Pharmacol 1998 46 563 570 10.1046/j.1365-2125.1998.00836.x 9862245 PMC1873804

[B10] Russo MW Fried MW Side effects of therapy for chronic hepatitis C Gastroenterology 2003 124 1711 1719 10.1016/S0016-5085(03)00394-9 12761728

[B11] Oda K Ikehara Y Weakly basic amines inhibit the proteolytic conversion of proalbumin to serum albumin in cultured rat hepatocytes Eur J Biochem 1985 152 605 609 10.1111/j.1432-1033.1985.tb09238.x 3902474

[B12] Oda K Koriyama Y Yamada E Ikehara Y Effects of weakly basic amines on proteolytic processing and terminal glycosylation of secretory proteins in cultured rat hepatocytes Biochem J 1986 240 739 745 3493770 10.1042/bj2400739 PMC1147481

[B13] de Duve C de Barsy T Poole B Trouet A Tulkens P Van Hoof F Commentary. Lysosomotropic agents Biochem Pharmacol 1974 23 2495 2531 10.1016/0006-2952(74)90174-9 4606365

[B14] Poole B Ohkuma S Effect of weak bases on the intralysosomal pH in mouse peritoneal macrophages J Cell Biol 1981 90 665 669 10.1083/jcb.90.3.665 6169733 PMC2111912

[B15] Vincent MJ Bergeron E Benjannet S Erickson BR Rollin PE Ksiazek TG Seidah NG Nichol ST Chloroquine is a potent inhibitor of SARS coronavirus infection and spread Virol J 2005 2 69 10.1186/1743-422X-2-69 16115318 PMC1232869

[B16] Savarino A Boelaert JR Cassone A Majori G Cauda R Effects of chloroquine on viral infections: an old drug against today's diseases? Lancet Infect Dis 2003 3 722 727 10.1016/S1473-3099(03)00806-5 14592603 PMC7128816

[B17] Savarino A Gennero L Chen HC Serrano D Malavasi F Boelaert JR Sperber K Anti-HIV effects of chloroquine: mechanisms of inhibition and spectrum of activity Aids 2001 15 2221 2229 10.1097/00002030-200111230-00002 11698694

[B18] Di Trani L Savarino A Campitelli L Norelli S Puzelli S D'Ostilio D Vignolo E Donatelli I Cassone A Different pH requirements are associated with divergent inhibitory effects of chloroquine on human and avian influenza A viruses Virol J 2007 4 39 10.1186/1743-422X-4-39 17477867 PMC1878474

[B19] Sperber K Chiang G Chen H Ross W Chusid E Gonchar M Chow R Liriano O Comparison of hydroxychloroquine with zidovudine in asymptomatic patients infected with human immunodeficiency virus type 1 Clin Ther 1997 19 913 923 10.1016/S0149-2918(97)80045-8 9385480

[B20] Paton NI Aboulhab J Hydroxychloroquine, hydroxyurea and didanosine as initial therapy for HIV-infected patients with low viral load: safety, efficacy and resistance profile after 144 weeks HIV Med 2005 6 13 20 10.1111/j.1468-1293.2005.00259.x 15670247

[B21] Mosmann T Rapid colorimetric assay for cellular growth and survival: application to proliferation and cytotoxicity assays J Immunol Methods 1983 65 55 63 10.1016/0022-1759(83)90303-4 6606682

[B22] Bartosch B Dubuisson J Cosset FL Infectious hepatitis C virus pseudo-particles containing functional E1-E2 envelope protein complexes J Exp Med 2003 197 633 642 10.1084/jem.20021756 12615904 PMC2193821

[B23] Drummer HE Maerz A Poumbourios P Cell surface expression of functional hepatitis C virus E1 and E2 glycoproteins FEBS Lett 2003 546 385 390 10.1016/S0014-5793(03)00635-5 12832074

[B24] Thorens B Vassalli P Chloroquine and ammonium chloride prevent terminal glycosylation of immunoglobulins in plasma cells without affecting secretion Nature 1986 321 618 620 10.1038/321618a0 3086747

[B25] Dille BJ Johnson TC Inhibition of vesicular stomatitis virus glycoprotein expression by chloroquine J Gen Virol 1982 62 Pt 1 91 103 10.1099/0022-1317-62-1-91 6290597

[B26] Tsai WP Nara PL Kung HF Oroszlan S Inhibition of human immunodeficiency virus infectivity by chloroquine AIDS Res Hum Retroviruses 1990 6 481 489 10.1089/aid.1990.6.481 1692728

[B27] Savarino A Lucia MB Rastrelli E Rutella S Golotta C Morra E Tamburrini E Perno CF Boelaert JR Sperber K Cauda R Anti-HIV effects of chloroquine: inhibition of viral particle glycosylation and synergism with protease inhibitors J Acquir Immune Defic Syndr 2004 35 223 232 10.1097/00126334-200403010-00002 15076236

